# Mixed plantations of *Metasequoia glyptostroboides* and *Bischofia polycarpa* change soil fungal and archaeal communities and enhance soil phosphorus availability in Shanghai, China

**DOI:** 10.1002/ece3.7532

**Published:** 2021-05-27

**Authors:** Weiwei Zhang, Wen Liu, Shanwen He, Qingchu Chen, Jigang Han, Qingfei Zhang

**Affiliations:** ^1^ Key Laboratory of National Forestry and Grassland Administration on Ecological Landscaping of Challenging Urban Sites Shanghai Academy of Landscape Architecture Science and Planning Shanghai China; ^2^ Shanghai Engineering Research Center of Landscaping on Challenging Urban Sites Shanghai China; ^3^ Shanghai Chenshan Botanical Garden Shanghai China

**Keywords:** archaeal community, bacterial community, fungal community, mixed plantation, pure plantation

## Abstract

Soil degradation has been found in urban forests in Shanghai, especially in the pure plantations. Mixed plantations are considered to improve soil quality because they can stimulate organic matter cycling and increase soil carbon and nutrient content. Although soil microbes play crucial roles in regulating soil biogeochemical processes, little is known about how mixed plantations affect soil microbial communities, including bacteria, archaea, and fungi. Here, we evaluated soil chemical properties, abundances and compositions of soil bacterial, archaeal, and fungal communities, and enzyme activities in pure and mixed *Metasequoia glyptostroboides* and *Bischofia polycarpa* plantations, located in Shanghai, China. The results showed that soil available phosphorus content in the mixed plantation of *M. glyptostroboides* and *B. polycarpa* was significantly higher than that in pure plantations, while no significant difference was observed in the content of soil organic carbon, total and available nitrogen, total and available potassium among the three studied plantations. We found higher fungal abundance in the mixed plantation, when compared to both pure plantations. Moreover, fungal abundance was positively correlated with the content of soil available phosphorus. No significant difference was found in the abundance and diversity of bacterial and archaeal community among the three studied plantations. A similarity analysis (ANOSIM) showed that mixed plantation significantly altered the community composition of archaea and fungi, accompanied with an increase of alkaline phosphatase activity. However, ANOSIM analysis of bacterial communities showed that there was no significant group separation among different plantations. Overall, results from this study indicated that fungal and archaeal communities were more sensitive to aboveground tree species than bacterial community. Moreover, mixed plantations significantly increased the activity of alkaline phosphatase and the content of soil available phosphorus, suggesting that afforestation with *M. glyptostroboides* and *B. polycarpa* is an effective way to alleviate phosphorus deficiency in urban forests in Shanghai, China.

## Graphical abstract

Fungal and archaeal communities were more sensitive to aboveground tree species than bacterial community. Mixed plantations significantly increased the activity of alkaline phosphatase and the content of soil available phosphorus, suggesting that afforestation with *Metasequoia glyptostroboides* and *Bischofia polycarpa* is an effective way to alleviate phosphorus deficiency in urban forests in Shanghai, China.

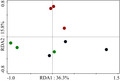

## 1 INTRODUCTION

Urban forest has undergone rapid development in China over the last three decades, especially in the highly developed areas along the East Coast (Wang et al., 2013). Urban forest is becoming an important component of forest ecosystems in China. As one of the most urbanized and developed cities in China, Shanghai showed an increase of urban forest coverage from 3% in the 1990s to 14% in 2014 (National Forestry & Grassland Administration, 2019). Thus, urban forest provides increasingly profound ecosystem services at a regional and national scale, such as maintaining biodiversity, reducing urban heat island effect, and cleaning air and water (Endreny, 2018; Millward et al., 2014; Xiao & McPherson, 2002). However, most of the Chinese urban forests, and especially those established during recent years, are pure plantations. Similar to other monoculture system, pure urban forest stands are facing many problems, such as a decline in ecosystem stability due to soil degradation and higher susceptibility to pests and pathogens (Endreny, 2018; Liu & Li, 2010; Steenberg et al., 2017). In addition, these pure stands have a negative impact on the landscape esthetic (Liu et al., 2004). To resolve these problems and enhance the ecological function of urban forest, afforestation with mixed species has been gradually adopted when establishing new plantations (Liu et al., 2004; Ordóñez & Duinker, 2014).

Given that soil microbes provide crucial roles in soil ecosystem processes, such as the decomposition of the soil organic matter and the mineralization of nutrients, increasing efforts have been made toward better understanding the effects of aboveground tree species on soil microbial biomass and community composition (Gunina et al., 2017; Pereira et al., 2019; Rachid et al., 2013; Wu, Zhang, et al., 2019). As reported, afforestation with mixed species may induce higher biomass and diversity of soil microbes than those in pure plantations (Gunina et al., 2017; Pereira et al., 2019; Thoms et al., 2010; Wen et al., 2014). Recently, Pereira et al. (2017) reported that mixed‐species plantations could influence the community composition of soil bacteria down to 300 cm depth. However, most of these studies were focused on bacteria or fungi, the studies of archaeal community in soil are lacking. Archaea were originally thought to exist only in harsh environments and were often described as “extremophiles,” but we now know they are widely distributed and participate in the circulation of critical element in soil, such as ammonia oxidation (Aislabie & Deslippe, 2013). Thus, it is essential to investigate bacterial, fungal, and archaeal communities simultaneously, to elucidate microbial mechanisms that contribute to improve soil carbon (C) and nutrient dynamics in mixed‐species plantations.

Soil microbes participated in soil C and nutrient cycling by secreting extracellular enzymes, which are responsible for controlling different reactions and metabolic processes in the biogeochemical cycling of nutrient elements (Saha et al., 2008). Thus, extracellular enzyme activities have been used widely as sensitive indicators for assessing microbial functionality (Acosta‐Martinez et al., 2007; Phillips et al., 2014; Zhang et al., 2017). Mixed‐species plantations have been found to increase, decrease or have no effect on extracellular enzyme activities (Kooch & Bayranvand, 2017; Lucas‐Borja et al., 2012; Pereira et al., 2019). These contradictory results may be partly attributed to the distinct responses of soil microbes to aboveground tree species. Thus, identifying the effects of aboveground tree species on soil enzyme activities is an important step toward understanding the potential mechanism involved with soil ecological processes.

*Metasequoia glyptostroboides* and *Bischofia polycarpa* are important urban greening tree species in Shanghai, China (Wang et al., 2013). In this study, we evaluated the composition and function of soil bacterial, fungal, and archaeal communities in pure and mixed *M. glyptostroboides* and *B. polycarpa* plantations in Shanghai, China. We hypothesized that the mixed plantation would (a) increase the content of soil carbon and nutrients; (b) increase the abundance and diversity of soil bacterial, fungal, and archaeal communities, and change their composition; (c) stimulate soil extracellular enzyme activities. The results of this study would provide a better understanding of the soil microbial communities in mixed and pure plantations.

## 2 MATERIALS AND METHODS

### 2.1 Study site and sampling

The study was carried out at ecological public welfare forests, located in Chongming district, Shanghai, China (31°27′00″–31°51′15″N, 121°09′30″–121°54′00″E). The area is characterized by a subtropical monsoon climate with the mean annual temperature of 15.8°C and annual precipitation of 1,122 mm. The soil was developed on alluvial materials (Wang et al., 2013). Three independent stands were randomly chosen in this study. The latitude and longitude of the three stands were 31°37′33″N, 121°42′50″E, 31°37′50″N, 121°41′12″E, and 31°36′42″N, 121°43′42″E, respectively. The study stands were afforested with pure or mixed plantations in 2008. Three fixed 15 × 15 m plots were established in each stand, including (a) a pure *M. glyptostroboides* plantation, (b) a pure *B. polycarpa* plantation, and (c) a mixed plantation of the two species. Distances between plots were greater than 150 m. Soil from each plot was collected at a depth of 0–20 cm in November 2017. Soil samples were collected from 8 points at each sampled plot using a soil corer (2.5 cm diameter) and then mixed thoroughly. Impurities such as rocks, plant roots, and other objects were removed. Soil material was sieved through a 2‐mm mesh. Each sample was divided into three parts: One was stored at −20°C for DNA extraction, the second was stored at 4°C for enzyme assays, and the remaining soil was air‐dried to determine the chemical properties.

### 2.2 Determination of soil chemical properties

Soil pH was determined in a 1:2.5 soil/water (w/v) suspension using a pH meter. Soil organic carbon (SOC) content was determined using the dichromate oxidation method (Cui et al., 2019). Soil total nitrogen (N) content was determined by the Kjeldahl method (Bremner & Mulvaney, 1982). Soil total phosphorus (P) content was determined following H_2_SO_4_–HClO_4_ digestion (Olsen & Sommers, 1982). Soil total potassium (K) content was determined by flame photometry after digestion in nitric acid, perchloric acid, and hydrofluoric acid. Soil available N content was determined by the alkali hydrolysis and diffusion method (Cornfield, 1960). Soil available P and K content were measured using ICP‐MS (NexION 300X) and ICP‐OES (Optima 7000DV), respectively.

### 2.3 Total DNA extraction

Total DNA was extracted from 250 mg of fresh soil using the PowerSoil^®^ DNA Isolation Kit (MoBio Laboratories), according to the manufacturer instructions. DNA quality was checked by agarose gel (0.8%) electrophoresis (5 V cm^−1^, 45 min). The extracted DNA was stored at −80°C and used for later molecular procedures.

### 2.4 Real‐time PCR assay

Bacterial and archaeal abundances were estimated by assessing the 16S rRNA gene copy numbers using real‐time PCR, and fungal abundance was estimated by assessing the 18S rRNA gene copy numbers. Real‐time PCR was performed on a Light Cycler^®^ 96 System (Roche). Bacterial and archaeal 16S rRNA gene were quantified using the primers 338F/518R and 344F/915R, respectively (Guo et al., 2014; Muyzer et al., 1993). Fungal 18S rRNA gene was quantified using SSU‐0817F/SSU‐1196R primers (Borneman & Hartin, 2000). Real‐time PCR assays were carried out in a 25 μl reaction volume using SYBR^®^ Premix Ex Taq™ II (Takara). Amplification specificity was confirmed by melting curve analysis and gel electrophoresis of the amplified fragments. Triplicate reactions were performed for each DNA sample. Standard curves were obtained through 10‐fold serial dilutions of linearized plasmids, which contained the 16S rRNA or 18S rRNA gene fragments. Amplification efficiencies for the bacterial, archaeal, and fungal assays were 103%, 96%, and 93%, respectively. Data are shown as rRNA gene copy numbers per gram of dried soil.

### 2.5 Illumina amplicon sequencing

An aliquot of the extracted DNA from each sample was used as a template for amplification. The primers 338F and 806R were used to amplify the V3−V4 hypervariable region of the bacterial 16S rRNA gene (Cui et al., 2019; Huse et al., 2008). The primers 524F‐10‐ext and Arch958R‐mod were used to amplify the archaeal 16S rRNA gene (Pires et al., 2012). The primers ITS1F and ITS2R were used to amplify the ITS1 region of the fungal rDNA (Gardes & Bruns, 1993; White et al., 1990). Amplicon sequencing was performed on an Illumina MiSeq platform (Illumina).

### 2.6 Bioinformatic analysis

Raw fastq files were processed and analyzed using the QIIME pipeline (Caporaso et al., 2010). Chimeric sequences were identified and removed using UCHIME (Edgar et al., 2011). The qualified sequences were clustered into operational taxonomic units (OTUs) at 97% of sequence similarity using Uparse (Edgar, 2013). The singletons were removed before the downstream analyses. The taxonomy of representative sequences for each OTU was determined using an RDP classifier (Wang et al., 2007), against the Silva reference database (http://www.arb‐silva.de) for the 16S rRNA genes and the Unite reference database (http://unite.ut.ee/index.php) for the ITS. To equalize read sizes for their comparison among soil samples, the OTU tables were normalized to identical sequencing depth for further standardization analysis. The Shannon–Wiener index was calculated to evaluate the diversity of the soil microbial community using Mothur (Schloss et al., 2009).

### 2.7 Enzyme activities and soil basal respiration

We assayed the potential activities of four extracellular enzymes, including cellobiohydrolase, β‐1,4‐N‐acetyl‐glucosamidase, alkaline phosphatase, and phenol oxidase. Enzyme activity was expressed as μmol of products produced per hour per gram of dried soil. The activities of cellobiohydrolase, β‐1,4‐N‐acetyl‐glucosamidase, and alkaline phosphatase were determined using the substrates 4‐nitrophenyl‐β‐D‐cellobioside, 4‐nitrophenyl‐N‐acetyl‐β‐D‐glucosaminide, and *p*‐nitrophenol phosphate, respectively (Yang et al., 2013; Zhang et al., 2017). For cellobiohydrolase, 1 g soil was incubated for 4 hr (37°C) with 4 ml of universal buffer (pH 8.0) and 1 ml of the appropriate substrate solution. The reaction was terminated by adding 1 ml of 0.5 M CaCl_2_ and 4 ml of 0.5 M NaOH, and the absorbance of the filtrate was read at 410 nm. The activities of β‐1,4‐N‐acetyl‐glucosamidase were assayed in the same way, except that the reaction mixture was incubated for 1 hr. For alkaline phosphatase, soil was incubated at 37°C for 1 hr with 4 ml of universal buffer (pH 8.0), 0.2 ml of toluene, and 1 ml of the appropriate substrate solution. The activity of phenol oxidase was measured using L‐3,4‐dihydroxyphenylalanine (L‐DOPA) as the substrate (Saiya‐Cork et al., 2002). The reaction mixture contained 2 g soil, 8 ml of 10 mM DOPA, and 6 ml of universal buffer (pH 8.0). The reaction mixture was incubated for 20 min at room temperature; then, the reaction was terminated by centrifugation at 5,000 *g* for 5 min. The absorbance of the filtrate was read at 460 nm. Soil basal respiration was analyzed by trapping and measuring the evolved CO_2_ over a 24‐hr period at 25°C (Alef & Nannipieri, 1995). Briefly, soil samples (50 g) were moistened to ~70% of its field water‐holding capacity and then incubated in sealed containers using 10 ml of 0.1 M KOH as a base trap. Three containers without soil samples were used as controls. The evolved CO_2_ was adsorbed in KOH and measured by HCl (0.05 M) titration, using phenolphthalein as an indicator. Soil basal respiration was expressed as mg CO_2_–C kg^−1^ soil per day.

### 2.8 Statistical analyses

One‐way ANOVA was used to test the effects of forest type on soil chemical properties, abundance, and diversity of soil microbial community, and extracellular enzymatic activities. We further analyzed the correlations between the microbial abundance and Shannon index with soil attributes and extracellular enzymatic activities using Pearson correlation analysis. Statistical analyses were performed using SPSS 16.0 software (SPSS Inc.). Differences at *p* < 0.05 were regarded as statistically significant. Prior to the analysis, data were log‐transformed when necessary to meet assumptions of normality. To determine whether microbial communities differed according to the forest types, a similarity analysis (ANOSIM) was performed using the “vegan” package in R (http://www.r‐project.org). To identify the microbial taxa responsible for the community differentiation among the different forest types, we employed ANOVA on all of the genera at each collection date using STAMP software (Parks et al., 2014). The differential genera with false discovery rate‐corrected *p* values < 0.05 were identified as indicator genera (Benjamini & Hochberg, 1995). Redundancy analysis (RDA) was conducted based on the relative abundances of bacterial, fungal, and archaeal genera and the activities of extracellular enzymes (Canoco 5.0).

## 3 RESULTS

### 3.1 Soil chemical properties

**FIGURE 1:**
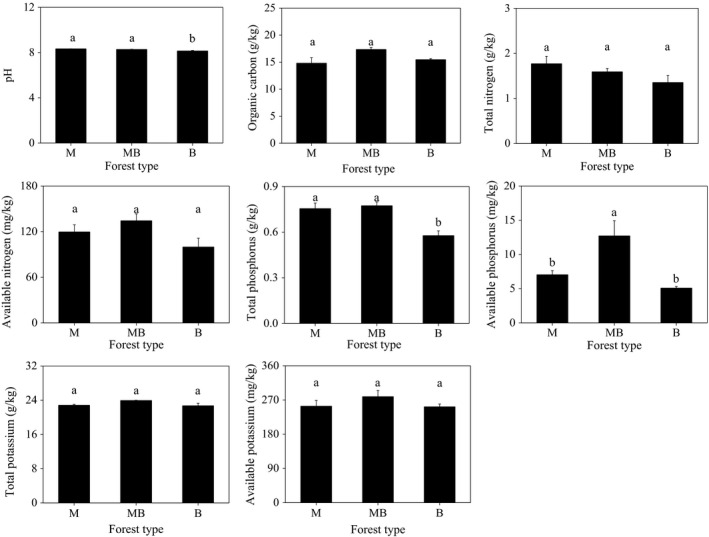
Soil chemical properties in pure and mixed *Metasequoia glyptostroboides* and *Bischofia polycarpa* plantations. Error bars indicate standard error of means (*n* = 3). M: the pure plantation of *M. glyptostroboides*; MB: the mixed plantation of *M. glyptostroboides* and *B. polycarpa*; B: the pure plantation of *B. polycarpa*

Soil available phosphorus of the soil in the mixed plantation of *M. glyptostroboides* and *B. polycarpa* were significantly higher than those in the pure plantations (Figure 1). Soil pH and total phosphorus content in the pure *B. polycarpa* plantation was significantly lower than the pure *M. glyptostroboides* plantation and the mixed plantation of *M. glyptostroboides* and *B. polycarpa*. Compared to the pure plantation soil, the mixed plantation of *M. glyptostroboides* and *B. polycarpa* did not affect soil organic carbon, total N, available N, total K, and available K (Figure 1).

### 3.2 Quantification of bacteria, archaea, and fungi

**FIGURE 2:**
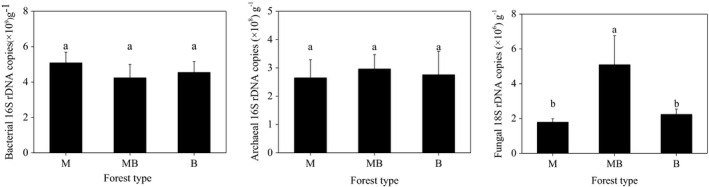
Abundance of bacterial 16S rRNA, archaeal 16S rRNA, and fungal 18S rRNA genes in pure and mixed *Metasequoia glyptostroboides* and *Bischofia polycarpa* plantations. Error bars indicate standard error of means (*n* = 3). M: the pure plantation of *M. glyptostroboides*; MB: the mixed plantation of *M. glyptostroboides* and *B. polycarpa*; B: the pure plantation of *B. polycarpa*

The abundance of fungi in the mixed plantation of *M. glyptostroboides* and *B. polycarpa* was significantly higher than that in the corresponding pure plantations (Figure 2). The abundance of fungal 18S rRNA gene in the mixed plantation of *M. glyptostroboides* and *B. polycarpa* was twofold greater than those found in the pure plantations of *M. glyptostroboides* and *B. polycarpa*. However, no differences were found in soil bacterial and archaeal abundances among the three studied plantations (Figure 2).

### 3.3 Microbial community composition

**FIGURE 3:**
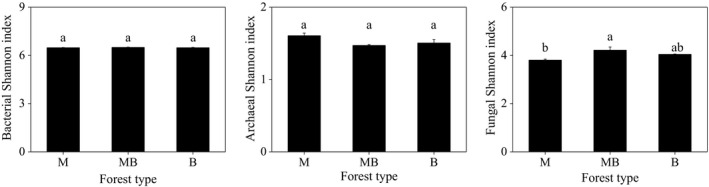
Shannon index for bacterial, archaeal, and fungal community in pure and mixed *Metasequoia glyptostroboides* and *Bischofia polycarpa* plantations. Error bars indicate standard error of means (*n* = 3). M: the pure plantation of *M. glyptostroboides*; MB: the mixed plantation of *M. glyptostroboides* and *B. polycarpa*; B: the pure plantation of *B. polycarpa*

Across all the samples, we obtained a total of 210,339, 278,073, and 555,750 high‐quality bacterial, archaeal, and fungal sequences, which were respectively grouped into 1,939, 80, and 1,347 OTUs at the 97% similarity. Shannon index for fungi in the mixed plantation of *M. glyptostroboides* and *B. polycarpa* was significantly higher than that in the pure plantation of *M. glyptostroboides* (Figure 3). In contrast, Shannon indices for bacteria and archaea were similar among the three plantations (Figure 3).

**FIGURE 4:**
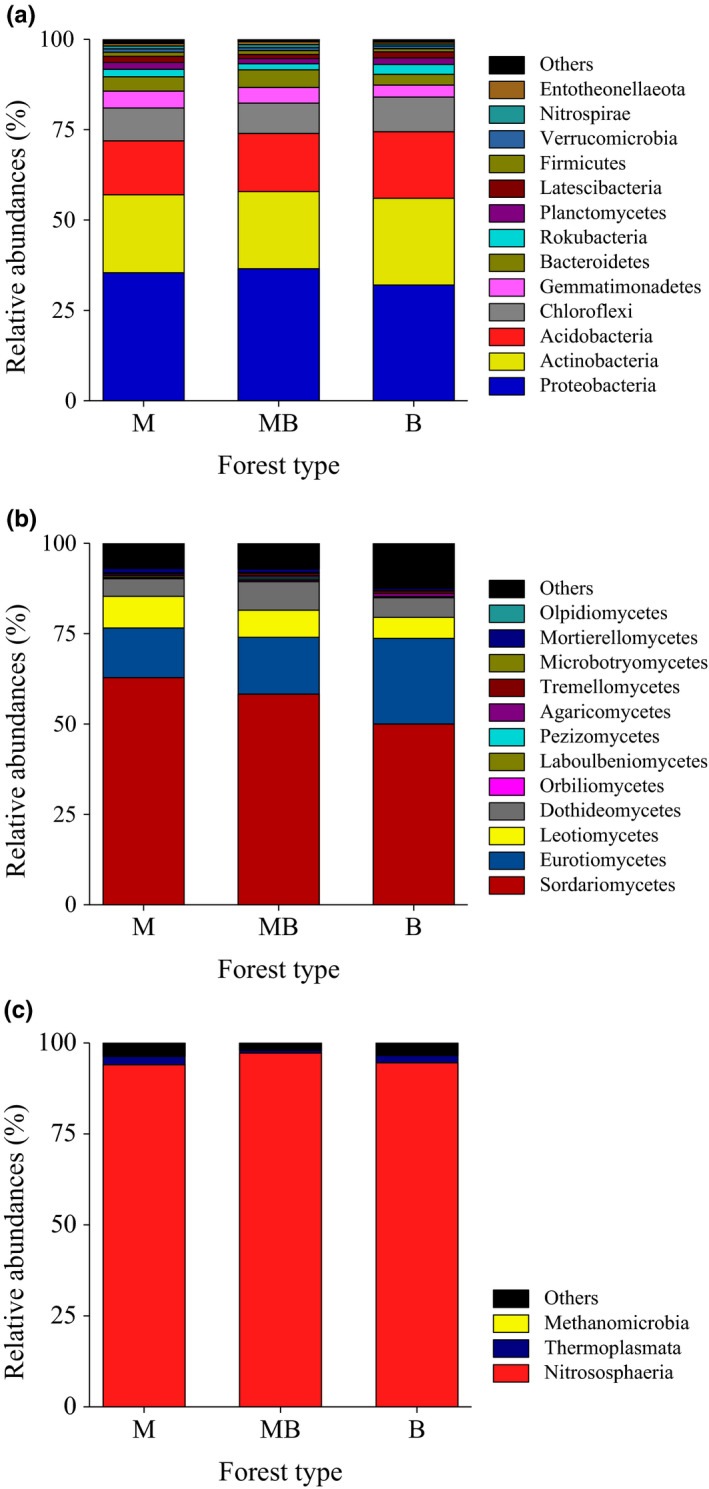
Relative abundances of bacteria (a), fungi (b), and archaea (c) at the phylum or class level in pure and mixed *Metasequoia glyptostroboides* and *Bischofia polycarpa* plantations. M: the pure plantation of *M. glyptostroboides*; MB: the mixed plantation of *M. glyptostroboides* and *B. polycarpa*; B: the pure plantation of *B. polycarpa*

Bacterial sequences were primarily composed of the phyla Proteobacteria (34.7%), Actinobacteria (22.3%), Acidobacteria (16.5%), and Chloroflexi (9.0%) (Figure 4). Relative abundances of these dominant bacterial phyla were similar among the three plantations. A total number of 437 bacterial genera were classified in this study. The relative abundances of these bacterial genera were similar to each other among the three plantations. ANOSIM analysis of bacterial communities based on the relative abundance of OTU showed that there was no significant group separation among different plantations (*R* = 0.1852; *p* = 0.161).

The most abundant archaeal phyla were Thaumarchaeota (95.3%) and Euryarchaeota (1.8%) (Figure 4). Relative abundances of these dominant archaeal phyla were similar among the three plantations. Only 12 archaeal genera were identified in this study, and significantly different was observed in the genus Candidatus *Nitrocosmicus*. ANOSIM analysis of archaeal communities based on the relative abundance of OTU showed that there was significant group separation among different plantations (*R* = 0.4074; *p* = 0.029).

The majority of fungal sequences belonged to the phyla Ascomycota (93.0%) and Basidiomycota (3.3%) (Figure 4). Relative abundance of the phyla Ascomycota in the pure plantation of *B. polycarpa* was significantly lower than the pure plantation of *M. glyptostroboides* and the mixed plantation of *M. glyptostroboides* and *B. polycarpa*. In contrast, the highest relative abundance of the phylum Basidiomycota was observed in the pure plantation of *B. polycarpa*. Fungal communities were dominated by Sordariomycetes (57.0%), Eurotiomycetes (17.7%), Leotiomycetes (7.4%), and Dothideomycetes (6.1%). Relative abundance of the class Sordariomycetes in the pure plantation of *B. polycarpa* was significantly lower than the pure plantation of *M. glyptostroboides* and the mixed plantation of *M. glyptostroboides* and *B. polycarpa*. In contrast, the highest relative abundance of the class Eurotiomycetes was observed in the pure plantation of *B. polycarpa*. Rare classes were also significantly affected by forest types, such as Cystobasidiomycetes, Glomeromycetes, Laboulbeniomycetes, Mortierellomycetes, and Zoopagomycetes. A total number of 403 fungal genera were classified in this study. *Neocosmospora* (8.3%), *Talaromyces* (7.5%), *Aspergillus* (6.7%), *Mycoarthris* (6.2%), and *Trichoderma* (5.4%) were the most abundant genera in this study. It should be noted that positive mixed effects were only founded in the rare fungal genera, such as *Titaea* and *Idriella*, the highest relative abundance of which were observed in the mixed plantation. ANOSIM analysis of fungal communities based on the relative abundance of OTU showed that there was significant group separation among different plantations (*R* = 0.5720; *p* = 0.004).

### 3.4 Enzymatic activity and soil basal respiration

**FIGURE 5:**
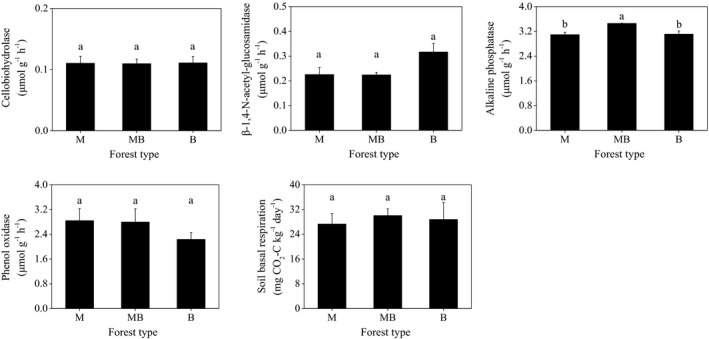
Activities of extracellular enzymes and soil basal respiration in pure and mixed *Metasequoia glyptostroboides* and *Bischofia polycarpa* plantations. Error bars indicate standard error of means (*n* = 3). M: the pure plantation of *M. glyptostroboides*; MB: the mixed plantation of *M. glyptostroboides* and *B. polycarpa*; B: the pure plantation of *B. polycarpa*

The activity of alkaline phosphatase was significantly higher in the mixed plantation than those in the pure plantations (Figure 5). Specifically, the mixed plantation, when compared with the pure plantations, showed increases in alkaline phosphatase activity of approximately 11%. In contrast, the activities of cellobiohydrolase, β‐1,4‐N‐acetyl‐glucosamidase, and phenol oxidase were similar among the three studied plantations (Figure 5). No significant difference was found in the soil basal respiration among the three studied plantations.

### 3.5 Relationships between the composition and function of microbial community and soil chemical properties

**FIGURE 6:**
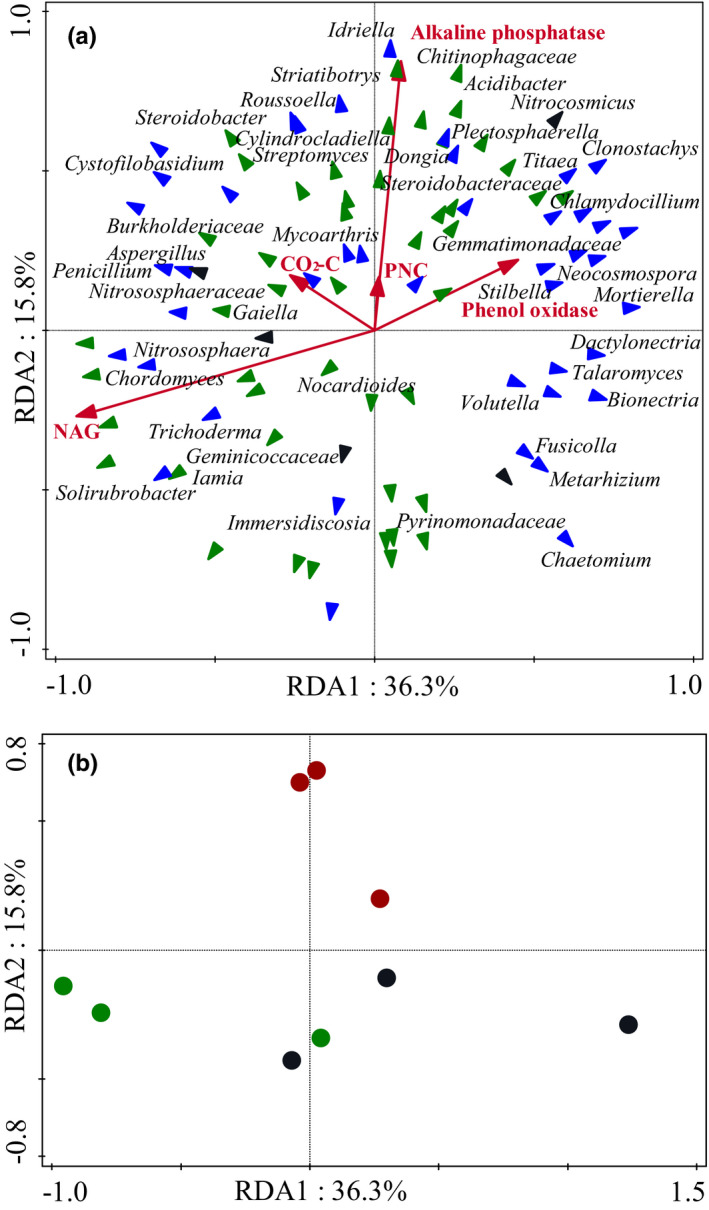
Redundancy analysis of the relative abundances of bacterial, fungal, and archaeal genera and enzymatic activities (a, b). Black circles: the pure *Metasequoia glyptostroboides* plantation; red circles: the mixed *M. glyptostroboides* and *Bischofia polycarpa* plantation; green circles: the pure *B. polycarpa* plantation. PNC: cellobiohydrolase; NAG: β‐1,4‐N‐acetyl‐glucosamidase

Pearson correlation analysis showed a strong positive correlation between fungal abundance and soil organic carbon, available P and K (Table 1). Archaeal abundance correlated positively with soil basal respiration (Table 1). No significant correlation was observed between bacterial abundance and soil chemical properties or enzyme activities. RDA analysis, based on the relative abundance of bacterial, archaeal, and fungal genera and extracellular enzyme activities, showed that the first and second axes extracted 55.60% and 16.56% of the explained variance, respectively (Figure 6). Moreover, the relative abundance of rare fungal genus *Idriella* and *Titaea*, and rare archaeal genus Candidatus *Nitrocosmicus* were positive related to the activity of alkaline phosphatase (Figure 6).

**TABLE 1:** Pearson's correlation test between microbial abundance and Shannon index with soil attributes and extracellular enzymatic activities

Soil	Microbial abundance			Microbial Shannon index		
	Bacteria	Archaea	Fungi	Bacteria	Archaea	Fungi
pH	ns	ns	ns	Ns	ns	ns
Soil organic carbon	ns	ns	0.730*	Ns	ns	ns
Total nitrogen	ns	ns	ns	ns	ns	ns
Available nitrogen	ns	ns	ns	ns	ns	ns
Total phosphorus	ns	ns	ns	ns	ns	ns
Available phosphorus	ns	ns	0.819*	ns	ns	ns
Total potassium	ns	ns	ns	ns	ns	ns
Available potassium	ns	ns	0.726*	ns	ns	ns
Cellobiohydrolase	ns	ns	ns	ns	ns	ns
Β‐1,4‐N‐acetyl‐glucosamidase	ns	ns	ns	ns	ns	ns
Alkaline phosphatase	ns	ns	ns	ns	ns	ns
Phenol oxidase	ns	ns	ns	ns	ns	ns
Soil basal respiration	ns	0.856*	ns	ns	ns	ns

ns means no significant difference.

* *p* < 0.05.

## 4 DISCUSSION

### 4.1 Effects of mixed tree species on soil chemical properties

Compared to monospecific plantations, afforestation with mixed tree species typically results in an increase in soil C and nutrient content and an improvement in soil quality (Wen et al., 2014). However, our study showed that the content of SOC, total and available N, and total and available K were not significantly affected by the forest types. These results contradict our hypothesis that the mixed tree species can increase the content of soil C and nutrients. A Previous study has showed that the mixed plantations sequestrated more soil organic carbon and nitrogen than pure plantations, and admixing effects enhanced with stand ages (Liu et al., 2017). Therefore, a possible explain for our results was that our study was conducted in young forests, and the soil C, N, and K content appeared to be less sensitive to above tree species composition during early stages of tree growth. In contrast, the mixed plantation of *M. glyptostroboides* and *B. polycarpa* exhibited significantly higher content of soil available P than pure plantations, which is consistent with other studies. Firn et al. (2007) has reported that the available P in the top soil was positively related to the aboveground tree species diversity. There are several potential reasons for the increase of soil available P in the mixed plantations, including a greater P inputs from litter and root, changes in root exudation (i.e., organic acids), and soil microbial activities (Forrester et al., 2005; Inagaki et al., 2011; Rachid et al., 2013). Compared to pure plantations, the mixed plantation significantly increased the content of soil available P rather than the soil C, N, and K content, which may be also relevant to the low available P level of the study sites. It should be noted that P is a very demanding nutrient in subtropical urban forests, and most urban forests in Shanghai were limited by P. Therefore, the significantly higher available P content in the soil found in the mixed plantations highlights the potential of afforestation with mixed tree species for alleviating phosphorus deficiency.

### 4.2 Effects of mixed tree species on microbial community and extracellular enzyme activity

In this study, the mixed plantation did not alter the abundance and diversity of archaea and bacteria but significantly increase the abundance and diversity of fungi. This finding is not consistent with studies of subtropical forest ecosystems showing that mixed plantations can increase both soil bacterial and fungal biomass compared to pure plantations (Huang et al., 2014; Lucas‐Borja et al., 2012). The ANOSIM test showed that bacterial community structure in the upper soil was not significantly altered by mixed plantations. In contrast, the community composition of fungi and archaea had significant responses to aboveground tree species. Our hypothesis that a mixed plantation can alter soil microbial communities was not fully confirmed. Previous study has demonstrated that both aboveground tree species and soil characteristics play vital roles in shaping soil microbial community (Pei et al., 2016). Soil bacterial community, including the abundance, diversity, and composition, was not significantly affected by the mixing of *M. glyptostroboides* and *B. polycarpa*, which may be due to the fact that most of bacteria can well adapt to various environments (Hemmat‐Jou et al., 2018; Sul et al., 2013; Zhang et al., 2017). The results indicated that bacteria appeared to be less sensitive to aboveground tree species than archaea and fungi in this study.

Phosphatase activity has been suggested as an indicator of P availability in soils (Chen et al., 2008). In this study, compared to the pure plantations, the higher activity of alkaline phosphatase observed under the mixed plantation indicated the greater mineralization of organic P under the mixed plantation. Our results confirmed that tree diversity could promote soil enzyme activities and improve soil P condition (Kooch & Bayranvand, 2017). It is well known that microorganisms play important roles in soil P transformation (Rachid et al., 2013). Fungi, especially saprotrophic fungi, had more dominant roles in the mobilization of organic P than bacteria via the exudation of phosphatases (Wu, Xiang, et al., 2019). As reported, the most abundant fungal genera observed in this study, including *Talaromyces*, *Aspergillus*, and *Trichoderma,* have the ability to product and release the phosphatases; and the activity of phosphatase increased with the fungal biomass (Della Mónica et al., 2018; Plante, 2007). In addition, previous study showed that Thaumarchaeota may play an unexplored role in biogeochemical cycling of river phosphorus (Hu et al., 2016), and archaea are more sensitive to phosphate depletion than bacteria and fungi (Ragot et al., 2016). The results in this study provided evidence that the increase in soil alkaline phosphatase activity in the mixed plantation may be associated with the increased fungal abundance and the changes in the community composition of soil fungi and archaea.

In this study, archaeal abundance showed a positive correlation with soil basal respiration, which was a major process that controlled the C loss from terrestrial ecosystem. This result suggested that archaea, instead of bacteria and fungi may play crucial roles in soil C mineralization in urban forests in Shanghai, China (Wang et al., 2020; Yu et al., 2021).

## 5 CONCLUSION

This study provides novel evidence that mixed plantations of *M. glyptostroboides* and *B. polycarpa* promote soil fungal abundance and change the community composition of fungi and archaea. Specifically, we showed that mixed plantations of *M. glyptostroboides* and *B. polycarpa* significantly increased the abundance and diversity of soil fungal community. In contrast, the abundance and diversity of bacterial and archaeal community were not significantly affected by the aboveground tree species. Moreover, mixed plantations significantly altered the community composition of fungi and archaea, but not bacteria. These results indicated that the response of fungal and archaeal community to aboveground tree species was more sensitive than bacterial community. Thus, composition and abundance of fungi and archaea in mixed plantations could be used as important parameters for assessing soil restoration in urban forests. In addition, mixed plantations significantly increased the activity of alkaline phosphatase and the content of soil available phosphorus. These results indicated that the mixed plantations may enhance soil P cycling, and afforestation with *M. glyptostroboides* and *B. polycarpa* proves to be an effective way to alleviate phosphorus deficiency in urban forests in Shanghai, China.

## CONFLICT OF INTEREST

The authors declare no conflicts of interest.

## AUTHOR CONTRIBUTIONS

**Weiwei Zhang:** Conceptualization (lead); Data curation (lead); Formal analysis (lead); Funding acquisition (supporting); Investigation (lead); Methodology (lead); Project administration (lead); Resources (lead); Software (lead); Supervision (lead); Validation (lead); Visualization (lead); Writing‐original draft (lead); Writing‐review & editing (lead). **Wen Liu:** Data curation (supporting); Methodology (supporting); Resources (supporting); Software (supporting). **Shanwen He:** Data curation (supporting); Formal analysis (supporting); Resources (supporting); Software (supporting). **Qingchu Chen:** Data curation (supporting); Formal analysis (supporting); Resources (supporting); Software (supporting). **Jigang Han:** Conceptualization (lead); Data curation (supporting); Funding acquisition (lead); Investigation (equal); Methodology (equal); Project administration (lead); Supervision (supporting); Validation (supporting); Visualization (supporting); Writing‐original draft (supporting); Writing‐review & editing (supporting). **Qingfei Zhang:** Conceptualization (equal); Formal analysis (equal); Funding acquisition (supporting); Investigation (equal); Project administration (supporting); Supervision (supporting); Validation (supporting); Writing‐original draft (supporting); Writing‐review & editing (supporting).

## Data Availability

The Illumina sequencing data were deposited to the NCBI Sequence Read Archive database (https://www.ncbi.nlm.nih.gov/sra) under accession number SRP266874, SRP266888, and SRP266995.
